# Exploring the synergy of enzymes, nutrients, and gene networks in rice starch granule biogenesis

**DOI:** 10.3389/fnut.2024.1448450

**Published:** 2024-10-23

**Authors:** Sunil Indrajit Warwate, Monika Awana, Swapnil S. Thakare, Veda Krishnan, Suresh Kumar, Haritha Bollinedi, Ajay Arora, Amitha Mithra Sevanthi, Mrinmoy Ray, Shelly Praveen, Archana Singh

**Affiliations:** ^1^Division of Biochemistry, ICAR-Indian Agricultural Research Institute, New Delhi, India; ^2^Division of Genetics, ICAR-Indian Agricultural Research Institute, New Delhi, India; ^3^Division of Plant Physiology, ICAR-Indian Agricultural Research Institute, New Delhi, India; ^4^ICAR-National Institute for Plant Biotechnology, New Delhi, India; ^5^Division of Forecasting and Agricultural Systems Modeling, ICAR-Indian Agricultural Statistics Research Institute, New Delhi, India

**Keywords:** rice endosperm, grain morphology, fatty acids, resistant starch, starch biogenesis, gene expression

## Abstract

**Introduction:**

Rice is a primary food source almost for more than 50% of the total world's population. Glycemic index (GI) is high in most of the rice varieties, limiting their consumption by diabetic and obese people. As a result, developing new rice varieties with low GI necessitates a thorough understanding of starch biogenesis gene expression and its interrelationship.

**Methods:**

A total 200 rice genotypes were analyzed for total starch content (TSC), amylopectin content (APC), and amylose content (AC). The clustering of these rice genotypes was done based on their AC. Further, these genotypes were categorized into three groups up to 10% amylose-low, 10–26% amylose-medium, and more than 26% amylose-high. Among them, six genotypes 1 from low AC (NJ-72), 2 from medium AC (UPRI-2003-18, PRR-126), and 3 from high AC (RNRM-7, Urvashi and Ananga) were selected. The genotypes selected from the medium and high AC groups were having 2% amylose variation among themselves respectively and they were further used to study the level of RS, protein content (PC), fatty acid (FA) profiles, and granule morphology along with low group sample.

**Results:**

Resistant starch (RS) content ranged from 0.33–2.75%, and fatty acid profiling revealed high levels of palmitic, linoleic, and oleic acids. The degree of crystallinity and APC% were found to be positively correlated. Ananga, the genotype with the highest RS, displayed compact starch granules. Further, NJ-72 showing low RS and Ananga with high RS were selected for investigation of enzymatic activities of starch biosynthesis, metabolites accumulation, and expressions of 20 starch biogenesis genes in developing endosperm. Starch branching enzymes (SBE) and starch synthase (SS) activities peaked at 13 days after anthesis (DAA), while starch debranching enzymes (DBE) were most active at 18 DAA. In Ananga, TSC, AC, APC, and RS levels progressively increased from 3 to 23 DAA. Ananga showed 1.25-fold upregulation of *granule-bound starch synthase I (GBSSI)* at 18DAA. Higher expressions of *SSI* and *SBEIIb* were observed in NJ-72 at 13DAA. *PUL2* was predominantly expressed followed by *ISA1*. GBSSI was positively correlated with both AC and RS while *SS, SBE*, and *DBE* were positively related to APC.

**Conclusion:**

This research could lead to the development of rice varieties with improved nutritional qualities, such as higher RS content, which is beneficial for human health due to its role in lowering glycemic response and promoting gut health. Additionally, the study provides insights into how the modulation of key genes and enzymes can affect starch composition, offering strategies to breed rice varieties tailored for specific dietary needs or industrial applications.

## 1 Introduction

Globally, 537 million adults (20–79 years) are living with diabetes. By 2030, this number is expected to reach 643 million, and by 2045, it is expected to reach 783 million. In 2021, diabetes was responsible for 6.7 million deaths, and it caused at least 966 billion dollars in health expenditure (1). Starchy foods have a high glycemic index (GI). Rice is consumed globally by about 4 billion people. White rice provides 23% of the world's calorie supply (2). It is a rich source of carbohydrates, and most of the rice varieties have high GI thus resulting in high blood glucose levels (3). Long-term consumption of such food leads to type II diabetes, fatigue, obesity, etc. Starch is the primary storage component in cereals, accounting for up to 80% of all calories taken by humans. Amylose and amylopectin are two major components of starch. Starch's nutritional qualities are determined by the amount of its principal components, ratio of amylose to amylopectin, crystallinity degree, granule morphology, and some minor components. Based on digestibility, starch is categorized into rapidly digestible, slowly digestible, and resistant starch (RS) (4). RS is a component of starch that remains undigestible in the small intestine by amylases and ferments in the large intestine by enzymes in healthy individuals. Generally, RS is divided into five types: RS1, RS2, RS3, RS4, and RS5. These are determined by the inaccessibility, structure, retrogradation, or chemical modification of starch, be it naturally occurring or added to foods (5). The level of RS is also contributed by the starch granule morphology and the crystallinity of amylopectin. Starch granules are heterogeneous in morphological structure, and hence their compactness, shape, etc. may determine the rate of starch hydrolysis. Protein is the second major component of rice grain next to the starch; therefore, its content may help for RS. Because RS gets digested incompletely in the small intestine, it has many health benefits, as it acts as soluble fiber (6), improves insulin sensitivity, lowers blood glucose levels, improves short-chain volatile fatty acid levels, and prevents inflammatory bowel disease (7). Developing cereal crops with a high RS content is increasingly necessary to tackle the rapidly growing challenges of nutrition as a public health issue.

Starch biosynthesis involves starch synthesizing, branching (BE), and debranching (DBE) enzymes that play a crucial role in controlling amylose and amylopectin ratio to regulate starch biosynthesis (8). Starch with high amylose content (AC) contributed to the formation of RS that reduces glycemic response (9). Starch synthesis enzymes are encoded by at least 7 groups of rice genome genes: two are for *granule-bound starch synthase (GBSSI)*, 8 for *starch synthase (SS)*, 6 for *ADP-glucose pyrophosphorylase (AGPase)*, 3 for *BE*, 2 for *phosphorylase (Pho)*, 4 for *DBE*, and 2 for *Disproportionating enzyme* (10). Individual isoforms of each class of enzyme have distinct relative activities that are tissue- and species-specific (11). AGPase mRNA and protein levels are relatively low during the initial stages of endosperm development, maximum at the middle, and again lower at maturation (12). In higher plants, SS has five subfamilies: GBSS, SSI, SSII, SSIII, and SSIV. There are two isoforms of GBSS: GBSSI and GBSSII. GBSSI, also referred to as WAXY protein is strongly linked to starch granules, catalyzes the amylose elongation in storage starch, and also accounts for a major proportion of overall GBSS activity (13, 14), whereas GBSSII is engaged in amylose biosynthesis in tissues not suitable for storage, such as leaves (15). In the opinion of Tetlow and Emes (16), as compared to SSI and SSII, SSIII generates longer and cluster-spanning chains (i.e., cluster-filling chains). SSIV, on the other hand, is involved in the initiation of starch granules rather than determining the amylopectin structure (17). In the rice genome, there is one *SSI* gene, three *SSII* genes (*SSIIa, SSIIb*, and *SSIIc*), two SSIII genes (*SSIIIa* and *SSIIIb*), and two *SSIV* genes (*SSIVa* and *SSIVb*). GBSSI and GBSSII each have a single gene (18). Studies showed that in rice, *OsSSIVa* exhibited lower and *OsSSIVb* exhibited higher expression levels in leaves and endosperm (10).

There are at least 3 SBE isoforms in rice endosperm (*OsSBEI, OsSBEIIa*, and *OsSBEIIb*). It was observed that a reduction in *OsSBEIIb* activity increases the AC in rice (19). DBEs in addition to SS and SBE play a crucial role in the production of semi-crystalline amylopectin, as DBE mutants showed lower amylopectin content (20). To date, two types of DBE have been identified in rice: isoamylase (ISA) and pullulanase (PUL). ISA has three copies, whereas the latter has only one. These enzymes' substrate specificities differed, with ISA debranching amylopectin and glycogen and PUL debranching pullulan and amylopectin (21). In compliance with Dinges et al. (22) in maize knockout mutants, endosperm and leaves require PUL to degrade starch normally. GBSS1 synthesizes amylose (23) while the combined activity of AGPase, SSs, SBEs, DBEs, PUL, and Phos are required for amylopectin biosynthesis (24). Basic experiments on alteration in the activities of important enzymes involved in starch, sucrose, and intermediary carbohydrate metabolism during endosperm development in the cereals are critical for identifying the role of essential enzymes concerning the synthesis of starch and also to unravel how their synthesis can be regulated throughout the endosperm development.

To understand the characteristics of grains in different developmental stages, we need to study and analyze the expression patterns of individual genes involved in starch synthesis. To our knowledge, scanty literature is available on starch granule biogenesis and expression analysis of genes involved in the developing endosperm of rice grains. The present study was carried out to unravel the dynamics of starch granule biogenesis and enzyme profiles for improved starch quality in rice. To this, we studied how the matrix components, starch granule morphology, and degree of amylopectin crystallinity may contribute to the production of RS. Further, we have investigated the expression profile of 20 starch biogenesis genes, their enzymatic activities, and metabolites in contrasting rice genotypes at different developing stages of rice endosperm. In the future, the current study might be helpful for people with diabetes or at risk of developing it, choosing the right genotype of rice can be an important first step toward diet-based diabetes prevention.

## 2 Material and methods

In the present study, grains of 200 mature rice genotypes were obtained from Division of Genetics, ICAR-Indian Agricultural Research Institute (IARI), New Delhi and total starch content (TSC), AC, and amylopectin content (APC) were carried out in the grains of these rice genotypes. Clustering of these rice genotypes was done using “cluster” package of R software into 3 clusters denoted as 1-blue colored, 2- yellow colored and 3- red colored (Supplementary Figure S1) based on their AC. Further, these genotypes were categorized into three groups as up to 10% amylose-low, 10%−26% amylose-medium, and more than 26% amylose–high. Among them six genotypes, 1 from low AC (NJ-72), 2 from medium AC (UPRI-2003-18, PRR-126), and 3 from high AC (RNRM-7, Urvashi and Ananga) were selected. The genotypes selected from medium and high AC group were having 2% amylose variation among themselves respectively and were further used to study the level of RS, protein content (PC), fatty acid (FA) profiles, and granule morphology along with low group sample.

Of these six genotypes, two contrasting RS genotypes (NJ-72; low RS and Ananga; High RS) were selected to study the gene expression levels, enzymatic activities, as well as levels of metabolites at different stages of endosperm development. Therefore, developing grains (2 biological and 3 technical replicates) of two contrasting rice genotypes, NJ-72 (low amylose), and Ananga (high amylose) were collected at five different endosperm developing stages [3 days after anthesis (DAA), 8DAA, 13DAA, 18DAA, and 23DAA] from the fields of Division of Genetics, ICAR-IARI), New Delhi, and stored at −80°C for further analysis. HiMedia (Delhi, India) and Sigma Aldrich (St. Louis Street, MO, USA) provided analytical-grade chemicals.

### 2.1 Estimation of metabolites

#### 2.1.1 Total starch

To estimate total starch content (TSC), Clegg's method (25) was used. One hundred mg of samples were finely ground using liquid nitrogen. The starch was extracted twice with hot 80% ethanol. The residue was treated with distilled water and perchloric acid (52%). At 25°C, centrifugation was performed for 10 min. A total of 100 mL of distilled water was added to the supernatant after it was pooled. Anthrone (0.2%) was added to a suitable aliquot, and absorbance at 620 nm was recorded. A glucose standard curve was used to calculate TSC and was expressed in percentage (%).

#### 2.1.2 Amylose content

Amylose content (AC) was estimated by Juliano's colorimetric method (26). One hundred mg of samples were finely grounded in liquid nitrogen, then 1 mL of 95% ethanol and 9 mL of 1 N sodium hydroxide were added. For 15 min, the tubes were placed in a boiling water bath. The volume was made to 100 mL with distilled water. In 100 mL volumetric flask, 5 mL of aliquot, 1 mL of 1 N acetic acid, and 2 mL of 0.2% iodine solution was added and kept in the dark for 20 min, and absorbance was noted down at 620 nm. A standard curve made from potato amylose (Sigma) was used to calculate the AC in samples and expressed in %.

#### 2.1.3 Amylopectin content

An Amylose/Amylopectin Megazyme assay kit was used to measure amylopectin content (APC). DMSO was used for defatting the 20 mg sample, and 95% ethanol was used to precipitate starch from the defatted sample. Then, 100 mM acetate-salt solution (pH 4.5) was added to it. Amylopectin was precipitated by centrifugation with a 4 mL solution of concanavalin A lectin. The amylose-containing supernatant was then hydrolyzed enzymatically into D-glucose and measured using glucose oxidase-peroxidase (GOPOD). As previously stated, the total starch in the acetate-salt solution was calculated separately. At 510 nm, the amount of amylopectin/amylose in the starch sample was calculated as a ratio of glucose-GOPOD absorbance in the concanavalin A precipitated sample supernatant.

#### 2.1.4 Resistant starch

The resistant starch (RS) was estimated using RS assay Kit (Megazyme International Ireland, Ltd., Bray, Ireland). One hundred mg powdered samples were digested with pancreatic α-amylase (10 mg/mL) containing amyloglucosidase (3 U/mL) for 16 h at 37°C with continuous shaking at 200 rpm. Centrifuged at 3,000 rpm for 10 min. RS pellet was washed twice with 50% ethanol, then suspended in 2 mL of 2 M KOH and kept on stirring in an ice-water bath for 10 min. Eight mL of sodium acetate buffer (1.2 M, pH 3.8), and 0.1 mL of amyloglucosidase (3 U/mL) were added. The tubes were vortexed and incubated at 50°C for 30 min. Centrifuged at 3,000 rpm for 10 min. 0.1 mL of supernatant was mixed with 3 mL of GOPOD and incubated at 50°C for 20 min. The absorbance was measured at 510 nm. RS was calculated as per the formula given in the kit itself and expressed in %.

#### 2.1.5 X-ray diffraction

Rice-powdered samples (1.2 g) were scanned with a Phillips X-ray diffractometer (PW 1710 diffractometer control, PW 1729 X-ray generator) with Automated Powder Diffractometer (APD) software using Ni-filtered Cu-Kα radiation (λ, 0.1542 nm) at a scanning speed of 1.5°2θ min^−1^ over a range of 4 to 50 °2θ. Using intensity (counts) and diffraction angle (°2θ) the crystallinity degree was calculated.

#### 2.1.6 Fatty acid profiling

Rice powdered samples weighing 1 g were dried overnight in an oven. Five mL of methanol and two drops of concentrated H_2_SO_4_ were added. The solution was incubated in a water bath at 65°C, cooled, and then mixed with 3 mL hexane. Fatty acid methyl esters (FAMEs) separated from the hexane layer. A hexane layer of 2 μL was injected into the GC system (DSQ-II model, Thermo Fisher Scientific, USA) at a flow rate of 10 mL/min. The stationary phase is the HP-5 MS capillary column, whereas the mobile phase is helium gas. Helium gas was injected at a rate of 1 mL/min. At 70 eV, the mass spectrometer was in electron impact mode. The composition of FA is represented as a proportion of total FA and expressed in %.

#### 2.1.7 Protein estimation

The protein content was estimated using Kjeldahl's method (27). In order to digest 500 mg of powered rice, sulfuric acid was used at a temperature of 350–380°C. Potassium sulfate and catalyst are added to improve digestion rates and efficiency. The sample is then cooled at 25°C, diluted with water, and transferred to a distillation unit after digestion. With the addition of NaOH during the distillation process, NH_4_^+^ is converted to NH_3_, which is captured by 2%−4% of aqueous boric acid in the receiver vessel. Titration is performed with sulfuric acid to determine nitrogen concentration. A conversion factor of 5.95 is applied to determine protein percentage.

#### 2.1.8 Granule morphology

Scanning electron microscope (Zeiss EVOMA10, Germany) was used to examine the granule morphology of six rice genotypes. Surgical blades were used to cut mature rice grains in half transversely. Specimens were gold coated for 30 s at 35 mA and then adhered on a circular aluminum stub with the cracks facing up using adhesive tape. After coating, the samples were photographed at an accelerator potential of 5 kV using SEM.

### 2.2 Activity assays of starch biogenesis enzymes in developing endosperm

#### 2.2.1 Starch synthase

Starch synthase (SS) activity was measured according to Leloir et al. (28). One g tissue was ground in 10 mL ice-cold extraction medium [0.05M *N*-2-hydroxyethyl piperazine-*N*′-ethanesulfonic acid pH 7, 10 mM EDTA, 5 mM dithiothreitol (DTT), and 1% polyvinylpyrrolidone]. The supernatant was collected after centrifugation for 20 min at 4°C at 15,000 g. The reaction mixture for the SS assay consisted of glycine buffer (0.08 M, pH 8.3), EDTA (4 mM), amylopectin (50 mg/mL), glutathione (40 mg/mL), and ADP-glucose (6 mM). An enzyme extract of 0.1 mL was added to tubes and incubated for 4 h in a shaking water bath at 37°C. A solution of phosphoenolpyruvate and pyruvate kinase (10 U) was added along with 0.2 mL dinitrophenyl hydrazine reagent, and it was incubated at 37°C for 15 min. A mixture of 0.2 mL NaOH (10 N) and 2 mL ethanol (95%) was added, and a 520 nm wavelength was used to measure the absorbance of brown color.

#### 2.2.2 Starch branching enzyme

Based on Hawker et al. method (29), starch branching enzyme (SBE) activity was measured. A sample of 0.5 g was ground in 2 mL of extraction buffer (50 mM 3-N-morpholino propane sulfonic acid pH 7.4, 2 mM MgCl_2_, 1 mM EDTA, and 2 mM DTT). The supernatant was collected after centrifugation at 15,000 g for 20 min at 4°C. The reaction mixture containing sodium citrate buffer (100 μM), amylase (300 μg), and 0.1 mL enzyme extract was incubated at 30°C for 15 min. The reaction was stopped by adding 0.5 mL of 2 N HCl followed by 1 mL iodine reagent, and using distilled water, the final volume was made to 5 mL. A wavelength of 590 nm was used to measure the absorbance.

#### 2.2.3 Starch debranching enzyme

The Fujita et al. method (30) was used to test the isoamylase (ISA) activity. One g of sample was homogenized in 3 mL of grinding solution (50 mM imidazole-HCl pH 7.4, 8 mM MgCl_2_, 50 mM 2-mercaptoethanol, and 12.5% glycerol). The supernatant was obtained after being centrifuged at 10,000 g for 2 min at 4°C. The reaction mixture containing MES-NaOH (50 mM, pH 6.5), CaCl_2_ (20 mM), rabbit liver glycogen (3 mg), and for 20 min, 0.1 mL of enzyme was incubated at 30°C. Tubes were placed in a boiling water bath for 1 min to terminate the reaction, and absorbance was measured at 520 nm.

Pullulanase (PUL) activity was determined following the protocol of Pullulanase/Limit-Dextrinase Assay Kit. The rice sample (250 mg) was ground in 12.5 mL of Buffer B (sodium acetate buffer, 100 mM, pH 5) and gently the slurry was stirred over about 15 min until the sample was completely dispersed. The solution was centrifuged (1,000 g, 10 min) and 0.1 mL aliquots of PULLG6 reagent solution were dispensed into test tubes and pre-incubated at 40°C for 5 min. PUL extract was also pre-incubated at 40°C for 5 min. To each tube containing PULLG6 reagent solution (0.1 mL), 0.1 mL of pre-equilibrated PUL extract was added directly to the bottom of the tube. Incubation was carried out at 40°C for 10 min, and the reaction was stopped by the addition of 3 mL of stopping reagent (2% Tris buffer solution, pH 9.0). Absorbance was read at 400 nm against distilled water, and PUL activity was calculated as per the formula provided by the manufacturer's protocol and expressed in PULLG6 unit/g.

By utilizing BSA as a standard and the Bradford method to assess total protein content, the specific activity (U/mg protein) for each enzyme was determined.

### 2.3 Expression profiling of starch biosynthesis genes

Total RNA was isolated from developing endosperms of both the genotypes (NJ-72 and Ananga) by the TRIzol method and cDNA was synthesized using a Verso cDNA synthesis kit (Fermentas). The coding sequences for all the starch biosynthesis-related genes were retrieved from NCBI and primers were designed for all the genes using Primer3Plus software (Supplementary Table S1). A quantitative real-time PCR mixture (20 μL) with 10 picomoles of forward and reverse primers was made using 1 μL of cDNA. The program includes initial denaturation (95°C) for 3 min followed by 39 cycles each of denaturation (94°C) for 20 s, annealing (52–65°C; depending on the Tm of specific primers) for 20 s, and extension (72°C) for 40 s. *Actin* and *18S rRNA* were used as reference genes. The mean of the replications was used to determine the Ct values for the reference genes and genes of interest to assess relative gene expression. The Pfaffl formula (Ratio = 2^−Δ*ΔCt*^) was used to calculate the fold change of the selected genes.

### 2.4 Statistical analysis

The correlation between 20 starch biogenesis genes with 4 metabolites (total starch, amylose, amylopectin, and RS) was developed by the “qgraph” package of R software. Phylogeny of starch biogenesis genes and their corresponding enzymes were studied by using Mega-X software. At a significance level of *p* < 0.05, Fisher's least significant difference was applied to assess the significant difference between the means. The information is given as mean standard deviation (SD).

## 3 Results

### 3.1 Matured rice genotypes showed different levels of metabolites

**Figure 1:**
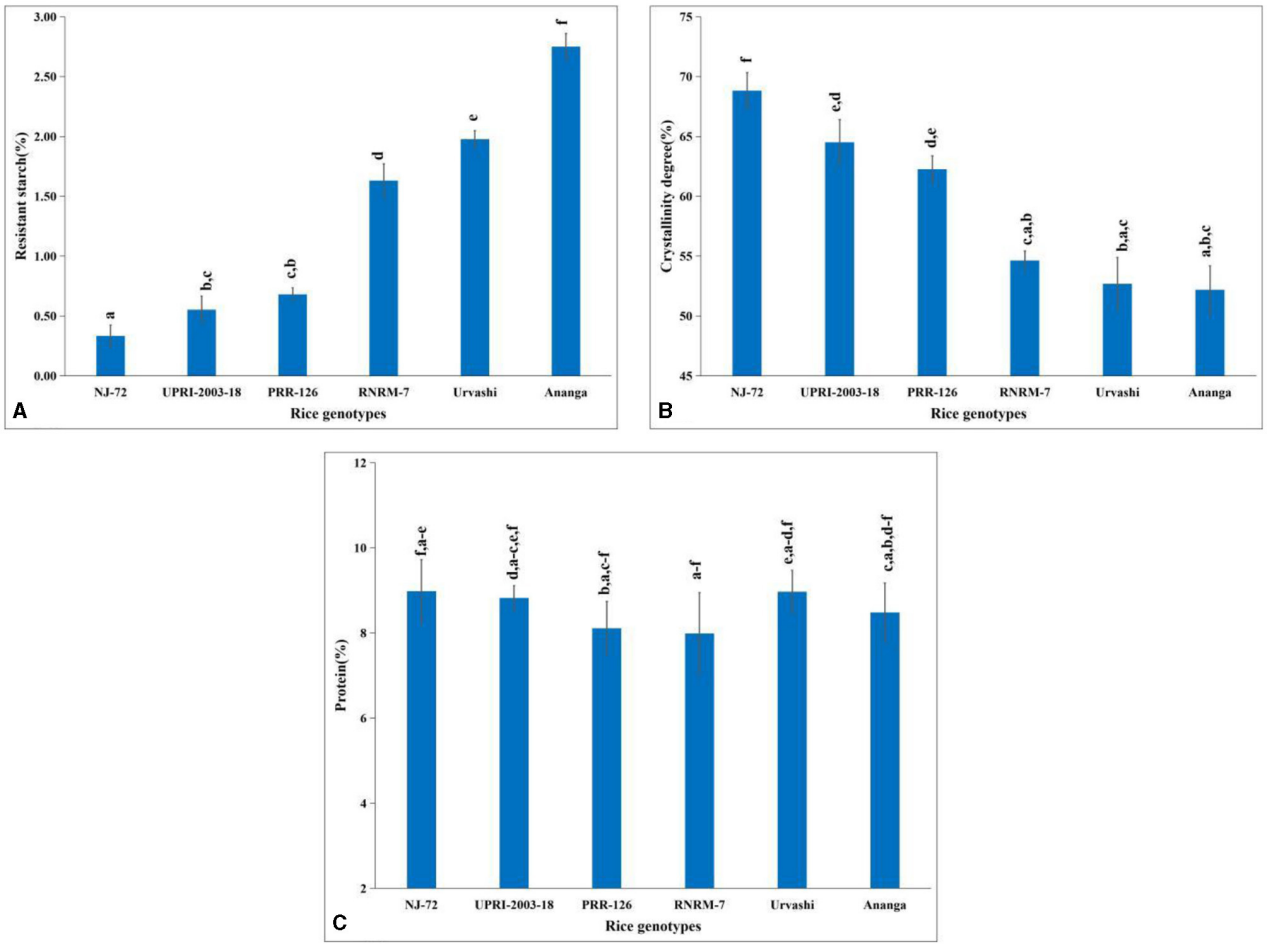
Change in metabolites concentration in contrasting six rice genotypes **(A)** resistant starch **(B)** amylopectin crystallinity and **(C)** protein content. Bar indicates ± SD. Different lowercase letters indicate a significant difference between mean at *p* < 0.05 [Fishers' least significant difference (LSD) test].

Rice starch and more precisely its principal components contribute to its nutritional quality, hence, we have estimated the percentage of TSC, AC, and APC in a total of 200 diversified mature rice genotypes. The variation in TSC depends on the growing location and varieties. In the current study, the TSC ranged from 67.31%−91.34% (Supplementary Table S2), where the Gull baber rice genotype had the highest TSC, while NJ-72 had the lowest. AC varied from 7.63%−33.68% in 200 matured rice genotypes (Supplementary Table S2). In NJ-72, the AC was lowest, while in Ananga the content was highest. The APC of 200 matured rice genotypes ranged from 39.57%−75.30% (Supplementary Table S2). Urvashi possessed the lowest amount of amylopectin, while Pant dhan 15 rice genotypes had the highest content. In the current study, the lowest RS was found in NJ-72 (0.33%), whereas Ananga (2.75%) has the highest RS (Figure 1A). RS content is 0.55, 0.68, 1.63, and 1.98% in UPRI-2003-18, PRR-126, RNRM-7, and Urvashi respectively.

### 3.2 Crystallinity degree

An amylopectin double helix is packed in a unit cell to ensure starch crystallinity. In the current study, genotypes with high AC have a lower percentage of crystallinity, and those with lower AC have a higher percentage of crystallinity. Amylopectin has a more crystalline structure than amylose. The percentage of crystallinity was 68.82, 64.50, 62.27, 54.63, 52.68, and 52.18 in NJ-72, UPRI-2003-18, PRR-126, RNRM-7, Urvashi, and Ananga respectively (Figure 1B). Compared with Urvashi and Ananga, the NJ-72 with a high crystallinity percentage might be due to its high APC.

### 3.3 Variation in protein content and fatty acid composition

The highest protein percentage was found in NJ-72 (8.98%) and the lowest was found in RNRM-7 (7.99%) (Figure 1C). The protein content is 8.82, 8.11, 8.97, and 8.48 percent in UPRI-2003-18, PRR-126, Urvashi, and Ananga, respectively.

The composition of different fatty acids (FA), including essential and non-essential FAs determines their nutritional importance. In the current study, FA profiling using GC-MS showed a total of 10 FAs in contrasting 6 rice genotypes (Table 1). These are myristic acid, palmitic acid, linoleic acid, oleic acid, linolenic acid, stearic acid, 11- eicosenoic acid, arachidonic acid, behenic acid, and lignoceric acid. Some of the FAs are not found in some genotypes. 11- Eicosenoic acid and linolenic acid were not detected in NJ-72 and RNRM-7 respectively. Behenic acid and lignoceric acid were not detected in UPRI-2003-18. Among the 10 FAs, palmitic acid, linoleic acid, and oleic acid were present in high amounts compared to the remaining 7 FAs. In the current study, palmitic acid and linoleic acid were found high in the high amylose genotypes of Ananga, Urvashi, and RNRM-7 followed by intermediate amylose genotypes, UPRI-2003-18 and PRR-126. The palmitic acid content varies from 33.69%−37.18%. Ananga has a 37.18% maximum palmitic acid while NJ-72 has a 33.69% minimum. Linoleic acid content varies from 28.42%−30.90%. Ananga has 30.90% of maximum linoleic acid while NJ-72 has a 28.42% minimum. Oleic acid was highest in the low amylose NJ-72 genotype, followed by intermediate amylose genotypes. Oleic acid content varies from 23.72%−34.96%. NJ-72 has a 34.96% maximum oleic acid, while Ananga has a 23.72% minimum. Although the content of myristic acid is low compared to the three major FAs (palmitic acid, linoleic acid, and oleic acid), it is found high in the high amylose rice genotypes, followed by genotypes with intermediate and low amylose. Myristic acid content varies from 1.07%−2.81%. Ananga has a 2.81% of maximum stearic acid while NJ-72 has a 1.07% minimum.

**Table 1:** The composition of individual fatty acids (FA) identified in contrasting six rice genotypes.

**S. No**.	**Fatty acids**	**NJ-72**	**UPRI-2003-18**	**PRR-126**	**RNRM-7**	**Urvashi**	**Ananga**
1	Myristic acid	0.92 ± 0.21	1.35 ± 0.18	2.32 ± 0.35	0.94 ± 0.30	1.42 ± 0.22	0.69 ± 0.19
2	Palmitic acid	28.20 ± 1.30	35.82 ±2.10	35.23 ± 1.80	31,881 ± 0.46	36.61 ± 2.01	37.68 ± 1.69
3	Linoleic acid	34.96 ± 0.99	18.52 ± 1.30	30.54 ± 1.20	27.24 ± 0.97	25.24 ± 0.58	22.72 ± 1.17
4	Oleic acid	33.96 ± 1.54	39.97 ± 1.02	25.95 ± 0.57	36.06 ± 1.79	26.81 ± 1.07	28.60 ± 0.89
5	Linolenic acid	0.55 ± 0.12	0.06 ± 0.01	0.14 ± 0.08	ND	4.56 ± 0.51	0.11 ± 0.07
6	Stearic acid	1.87 ± 0.09	3.31 ± 0.88	3.75 ± 0.71	1.46 ± 0.59	4.23 ± 1.04	5.81 ± 1.00
7	11- Eicosenoic acid	ND	0.28 ± 0.05	0.17 ± 0.02	0.17 ± 0.07	0.14 ± 0.11	1.07 ± 0.47
8	Arachidonic acid	0.12 ± 0.08	0.69 ± 0.32	0.46 ± 0.18	0.33 ± 0.05	0.52 ± 0.26	1.33 ± 0.42
9	Behenic acid	0.04 ± 0.02	ND	0.11 ± 0.04	0.05 ± 0.20	0.14 ± 0.08	0.74 ± 0.49
10	Lignoceric acid	0.05 ± 0.03	ND	0.2 ± 0.11	0.45 ± 0.33	0.33 ± 0.15	1.25 ± 0.93

ND represents not detected.

### 3.4 Starch granule morphology

**Figure 2:**
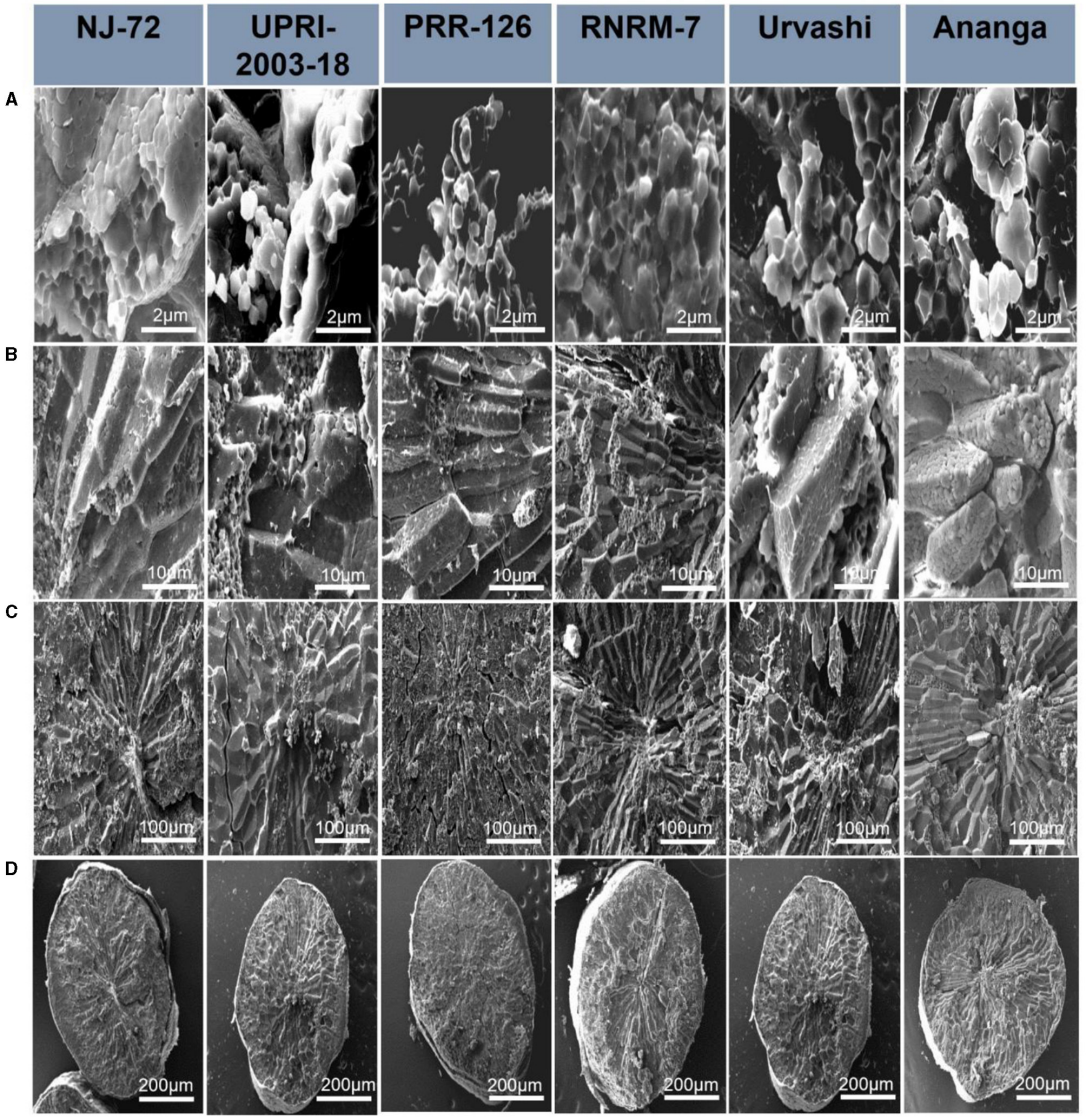
Scanning electron microscopy (SEM) of six rice genotypes having varying resistant starch content: NJ-72, UPRI-2003-18, PRR-126, RNRM-7, Urvashi and Ananga. Magnification obtained at **(A)** 2 μm, **(B)** 10 μm, **(C)** 100 μm, and **(D)** 200 μm.

Starch granules have differences in their compactness and shape, and hence these characteristics may influence starch hydrolysis. In the current study, the SEM micrograph of six rice genotypes (NJ-72, UPRI-2003-18, PRR-26, RNRM 7, Urvashi, and Ananga) having varying RS content is shown at 2, 10, 100, and 200 μM in Figure 2. The structural differences between genotypes were primarily observed at 2 μM, followed by 10 μM. With genotypes, the shape of starch granules varies significantly. NJ-72, a genotype with low amylose, exhibits loosely packed small, angular, or cube-shaped starch granules, while UPRI-2003-18 and PRR-126, genotypes with intermediate amylose, have loosely packed cube-shaped starch granules. A comparative analysis of the high amylose genotypes reveals that RNRM-7 and Urvashi have compound starch granules that are typically cubical, whereas Ananga has granules that are spherical with smooth surfaces.

### 3.5 Variation in metabolites at developing stages of endosperm

**Figure 3:**
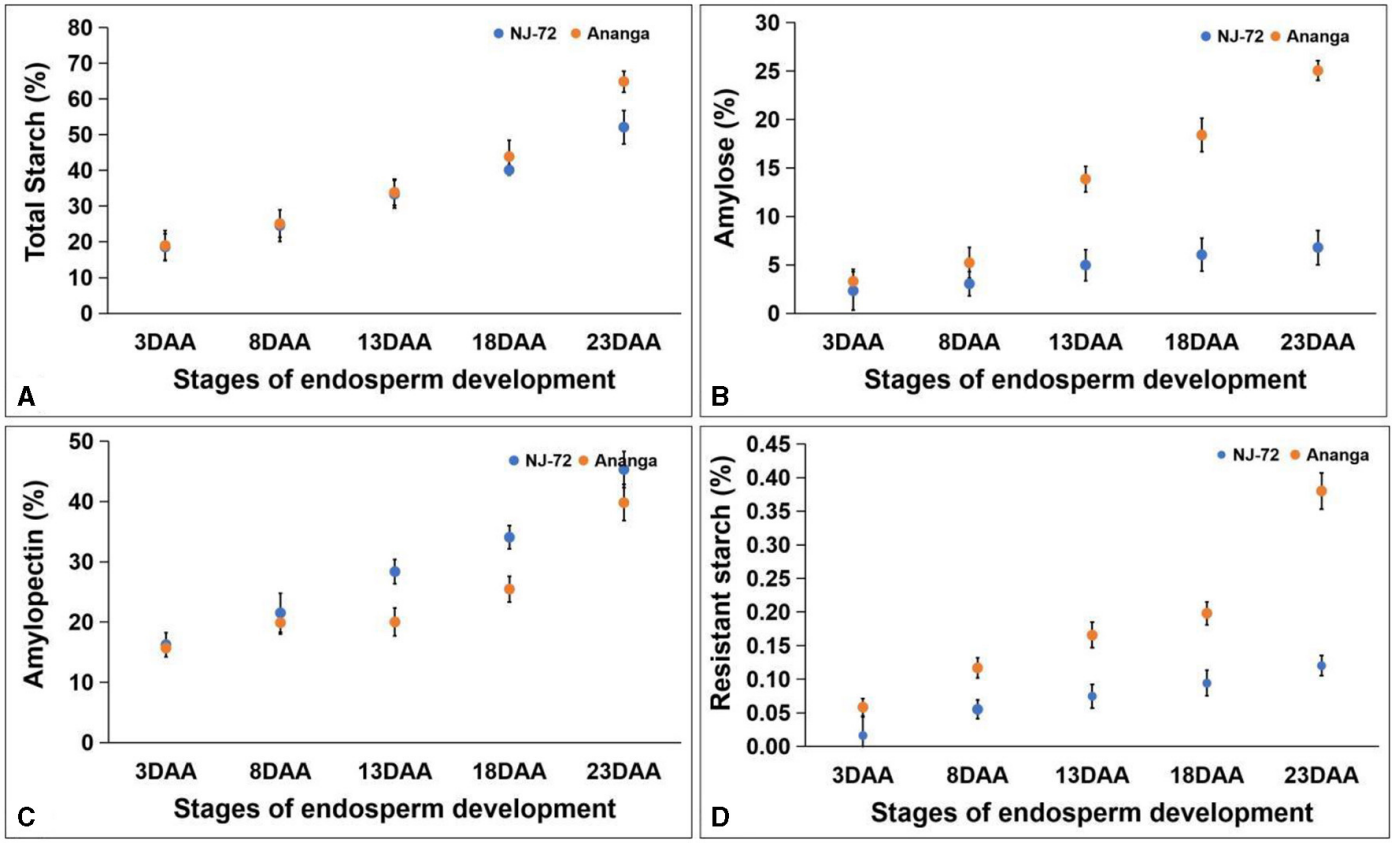
Change in metabolites with developing endosperm **(A)** total starch, **(B)** amylose, **(C)** amylopectin, and **(D)** resistant starch. DAA- days after anthesis. Bar indicates ± SD.

TSC in five developing stages of contrasting rice genotypes ranged between 18.58%−64.88%. TSC was maximum at 23DAA and minimum at 3DAA in both genotypes. Both the genotypes accumulated nearly equal amounts of TSC up to 13DAA, but beyond that Ananga showed more TSC in comparison to NJ-72. NJ-72 showed 50% and Ananga showed 64.88% TSC at 23DAA (Figure 3A). Amylose content varied from 2.33% to 25.05% in different developing stages of NJ-72 and Ananga (Figure 3B). It increased from 8DAA to 23DAA in both genotypes and was more in Ananga in all stages as compared to NJ-72. APC was found higher in NJ-72 in comparison to Ananga throughout the developing stages. NJ-72 showed 28.30%, 33.97%, and 45.19% while Ananga showed 19.85%, 25.28%, and 39.44% APC at 13, 18, and 23DAA respectively (Figure 3C). RS content in developing endosperms of NJ-72 and Ananga varied between 0.016%−0.380%. It increased from 3DAA to 23DAA in both the genotypes (Figure 3D) and was significantly higher in Ananga as compared to NJ-72 in all the stages. Ananga showed 0.4% while NJ-72 showed 0.1% RS content at 23DAA respectively.

### 3.6 Alteration in activities of starch biogenesis enzymes

**Figure 4:**
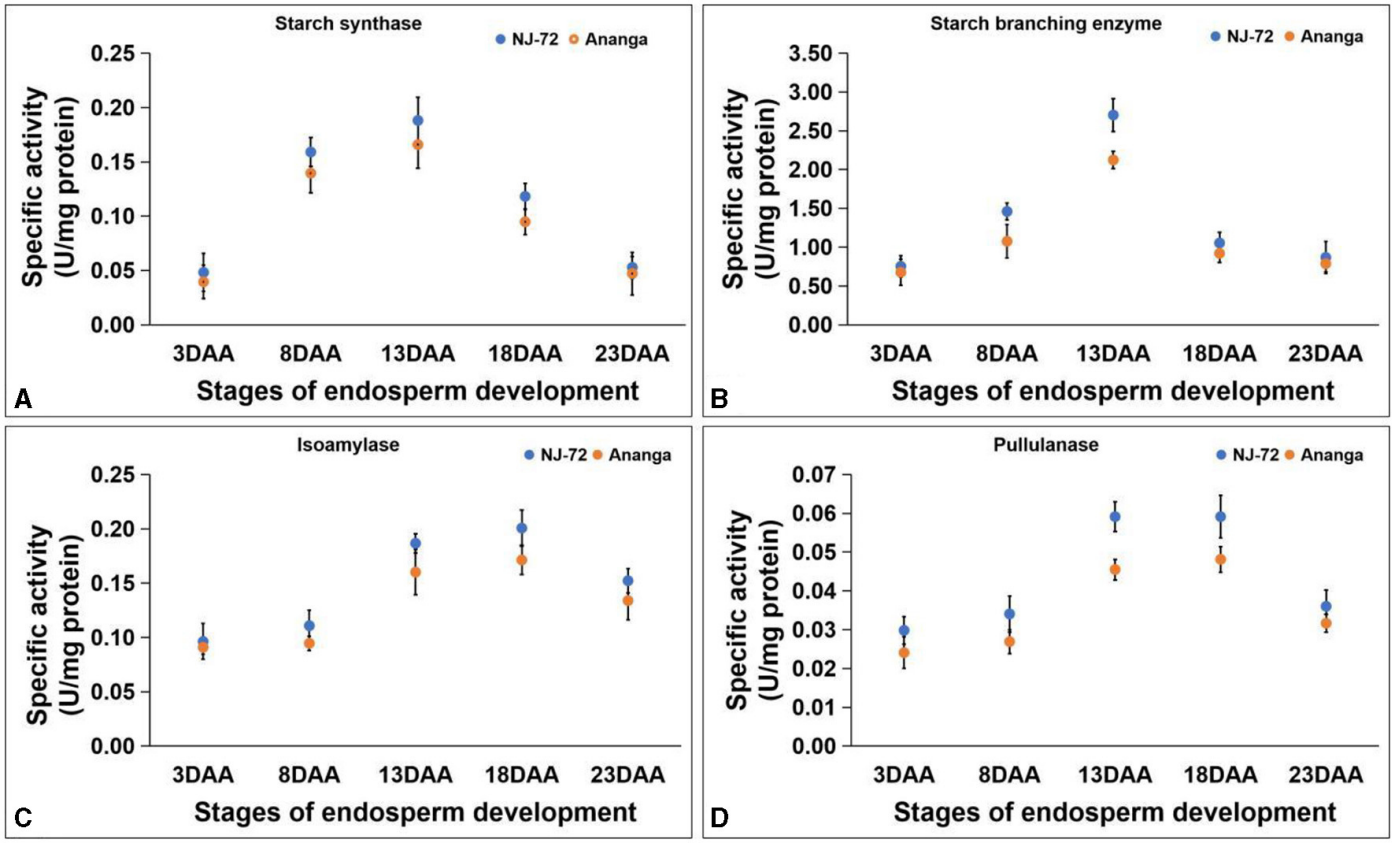
Change in activities of starch biogenesis enzymes with developing endosperm **(A)** starch synthase, **(B)** starch branching enzyme, **(C)** isoamylase and **(D)** pullulanase. Bar indicates ± SD.

SS activity in developing endosperms of NJ-72 and Ananga spans from 0.040–0.188 U/mg protein. NJ-72 showed more SS activity in all developing stages in comparison to Ananga. The maximum activity was observed at 13DAA in both genotypes. NJ-72 and Ananga exhibited 0.18 and 0.16 U/mg protein SS activity at 13DAA respectively (Figure 4A). SBE activity ranged from 0.676–2.703 U/mg protein in NJ-72 and Ananga. The activity was increased from 3DAA to 13DAA and after that decreased in both genotypes (Figure 4B). The maximum ISA and PUL activities were observed at 18DAA in both genotypes. The ISA activity ranged from 0.091–0.201 U/mg protein (Figure 4C) and PUL activity varied from 0.025–0.057 U/mg protein. At 18DAA, Ananga showed 0.057 and NJ-72 showed 0.043 U/mg protein PUL activity (Figure 4D). Among the DBEs, ISA activity was found to be significantly higher as compared to PUL in both the genotypes, which showed that ISA is a main DBE in starch biogenesis.

### 3.7 Differential expression of starch biogenesis genes in the developing endosperm

#### 3.7.1 ADP glucose pyrophosphorylase and granule-bound starch synthase

**Figure 5:**
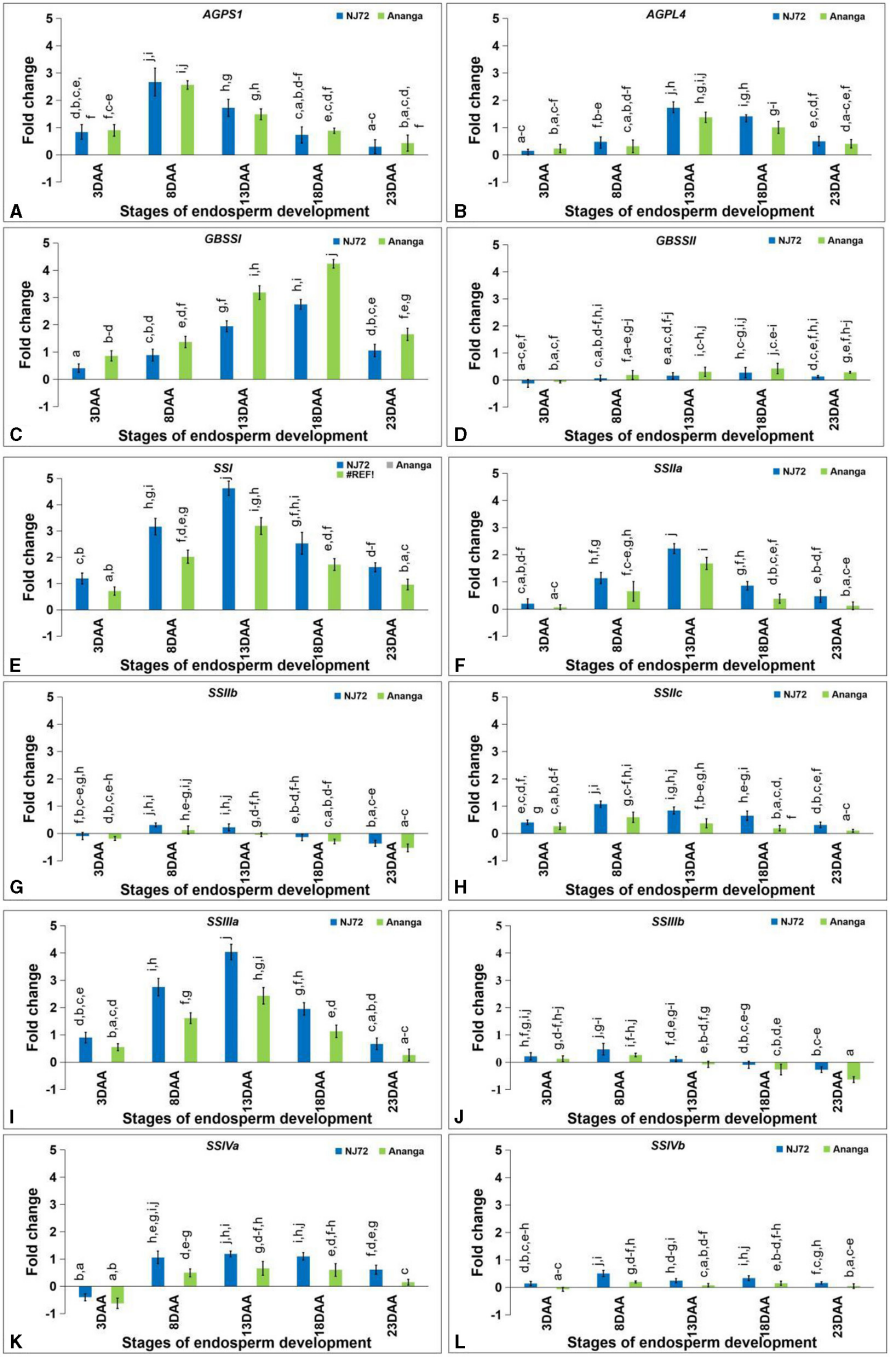
Differential expression of starch biogenesis genes in the developing endosperm of contrasting rice genotypes (NJ72 and Ananga) **(A)** *AGPS1*, **(B)** *AGPL4*, **(C)** *GBSSI*, **(D)** *GBSSII*, **(E)** *SSI*, **(F)** *SSIIa*, **(G)** *SSIIb*, **(H)** *SSIIc*, **(I)** *SSIIIa*, **(J)** *SSIIIb*, **(K)** *SSIVa*, and **(L)** *SSIVb*. Bar indicates ± SD. Different lowercase letters indicate significant difference between mean at *p* < 0.05 [Fishers' least significant difference (LSD) test].

The *AGPS1* expression level varied from 0.30–2.67 in NJ-72 and 0.43–2.76 in Ananga at different developing stages (Figure 5A). At 3DAA and 8DAA, *AGPL4* expression level was low, while at 13DAA it rapidly increased and then decreased toward 18DAA and 23DAA in both the genotypes (Figure 5B). At 13DAA, NJ-72 showed a 1.25-fold higher expression level of *AGPL4* in comparison to Ananga.

*GBSSI* expression was found maximum at 18DAA. It was more in Ananga in comparison to NJ-72 at all the stages. Ananga showed 1.25 and 1.63-fold more expression as compared to NJ-72 at 18 and 13DAA respectively (Figure 5C). *GBSSII* expression was downregulated at 3DAA in both the genotypes while upregulated in rest of the stages (Figure 5D). Ananga showed 2.3, 4, and 1.9-fold while NJ-72 showed 1.63, 2.3, and 1.52-fold more expression of *GBSSII* at 13, 18, and 23DAA respectively in comparison to their 8DAA stage.

#### 3.7.2 Starch synthases

The relative expression of *SSI* in the endosperm developmental stages ranged from 1.19–4.62 in NJ-72 and 0.71–3.19 in Ananga. The maximum expression was observed at 13DAA in both genotypes. NJ-72 showed a 1.44-fold upregulation of *SSI* in comparison to Ananga at 13DAA (Figure 5E). In the middle stage, the higher expression of *SSI* may be attributed to its role in elongating shorter chains (DP 6–10) of starch. The *SSIIa* expression was lower at the initial (3DAA) and final (23DAA) stages. At the middle stages, NJ-72 showed 1.8, 1.32, and 2.3-fold upregulation of *SSIIa* expression at 8, 13, and 18DAA respectively, in comparison to Ananga (Figure 5F). In all the developing endosperm stages, Ananga showed downregulation of *SSIIb* except at 8DAA (Figure 5G). NJ-72 showed upregulation of *SSIIb* at 8 and 13DAA while at other stages it was downregulated. NJ-72 showed 2.6-fold upregulation of *SSIIb* at 8DAA as compared to Ananga. The relative expression of *SSIIc* in NJ-72 ranged from 0.31–1.07, while in Ananga it ranged from 0.1–0.59 (Figure 5H). *SSIIc* was highly expressed at 8DAA in both genotypes. NJ-72 showed more upregulation at all stages in comparison to Ananga. The *SSIIIa* expression increases initially from 3DAA to 13DAA and then decreases at later stages in both genotypes (Figure 5I). NJ-72 showed 1.64, 1.71, 1.66, 1.73, and 2.58-fold upregulation of *SSIIIa* expression as compared to Ananga at 3, 8, 13, 18, and 23DAA respectively. NJ-72 and Ananga showed upregulation of *SSIIIb* expression at 3 and 8DAA while downregulation at later stages except NJ-72 at 13DAA (Figure 5J). The expression level of *SSIIIb* was less in Ananga as compared to NJ-72 at all developing stages. The *SSIVa* expression was downregulated at 3DAA in both genotypes. The *SSIVa* expression level ranged from 0.61–1.19 in NJ-72 and 0.15–0.66 in Ananga from 8DAA to 23DAA (Figure 5K). The expression level of *SSIVb* was found upregulated at all the stages in NJ-72, whereas in Ananga downregulation was observed at 3DAA and upregulation at the rest of the stages. At 23DAA, the *SSIVb* gene was not detected in Ananga (Figure 5L). NJ-72 showed 2.6, 3.1, and 2.3-fold upregulation of *SSIVb* gene at 8, 13, and 18DAA respectively in comparison to Ananga.

#### 3.7.3 Starch branching genes

**Figure 6:**
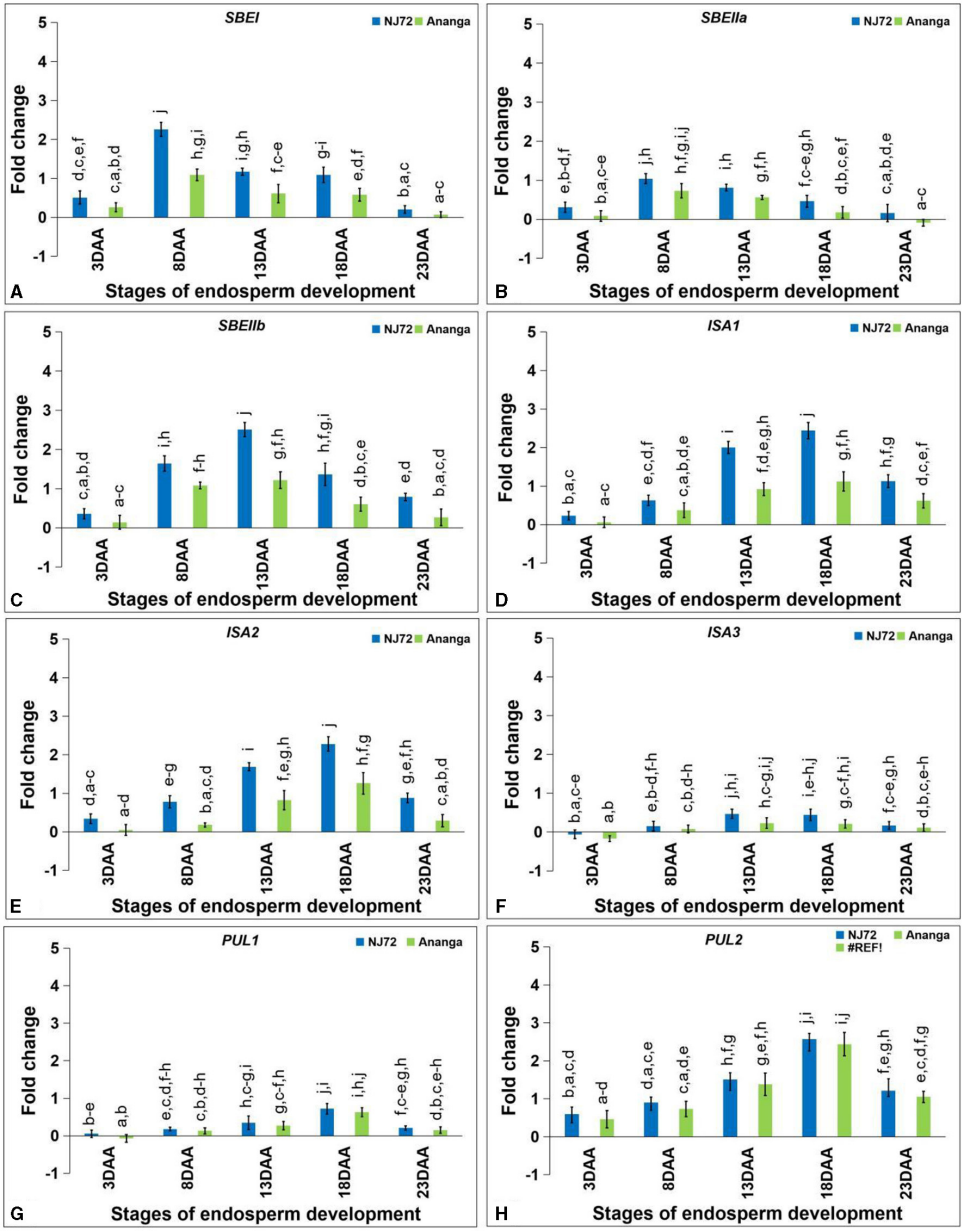
Differential expression of starch branching **(A)** *SBEI*, **(B)** *SBEIIa* and **(C)** *SBEIIb* and debranching **(D)** *ISA1*, **(E)** *ISA2*, **(F)** *ISA3*, **(G)** *PUL1*, and **(H)** *PUL2* genes in the developing endosperm of contrasting rice genotypes (NJ-72 and Ananga). Bar indicates ± SD. Different lowercase letters indicate significant difference between mean at *p* < 0.05 [Fishers' least significant difference (LSD) test].

The expression level of *SBEI* and *SBEIIa* was found maximum at 8DAA, while *SBEIIb* showed maximum expression at 13DAA. *SBEI* expression in NJ-72 showed 2.07, 1.91, and 1.88-fold variation at 8, 13, and 18DAA respectively as compared to Ananga (Figure 6A). *SBEIIa* expression in NJ-72 and Ananga showed 1.3-fold upregulation at 8DAA in comparison to 13DAA (Figure 6B). Ananga showed 2.57, 1.52, 2.06, 2.27, and 2.93-fold downregulation in *SBEIIb* gene level at 3, 8, 13, 18, and 23DAA respectively, as compared to NJ-72 at respective stages (Figure 6C).

#### 3.7.4 Starch debranching genes

Starch DBE, *ISA* (*ISA1, ISA2*, and *ISA3*) was increased from 3 to 18DAA and then decreased at 23DAA. NJ-72 showed more expression of *ISA* isoforms as compared to Ananga. The relative expression of *ISA1* was 2.2-fold higher in NJ-72 as compared to Ananga at 18DAA (Figure 6D). Ananga showed less expression of *ISA2* at all developing stages in comparison to NJ-72 (Figure 6E). The relative expression of *ISA2* ranged from 0.05–1.26 in Ananga and 0.34–2.28 in NJ-72 at all developing stages. The relative expression of *ISA3* was downregulated at 3DAA while upregulated at the rest of the stages in both genotypes (Figure 6F). At 13DAA, NJ-72 showed 2.04 upregulation of *ISA3* in comparison to Ananga.

The *PUL* gene expression was maximum at 18DAA. *PUL1* expression was low at the initial stages. NJ-72 showed more expression in comparison to Ananga. NJ-72 showed 2, 4, and 1.2-fold more upregulation of the *PUL1* gene at 13, 18, and 23DAA respectively, in comparison to its expression at 8DAA (Figure 6G). The relative expression of *PUL2* was higher in NJ-72 at all developing stages as compared to Ananga but the difference between the two genotypes concerning *PUL2* expression was much less (Figure 6H). At 18DAA, NJ-72 showed a 1-fold upregulation of the *PUL2* gene in comparison to Ananga.

### 3.8 Gene to metabolite correlation and phylogeny

**Figure 7:**
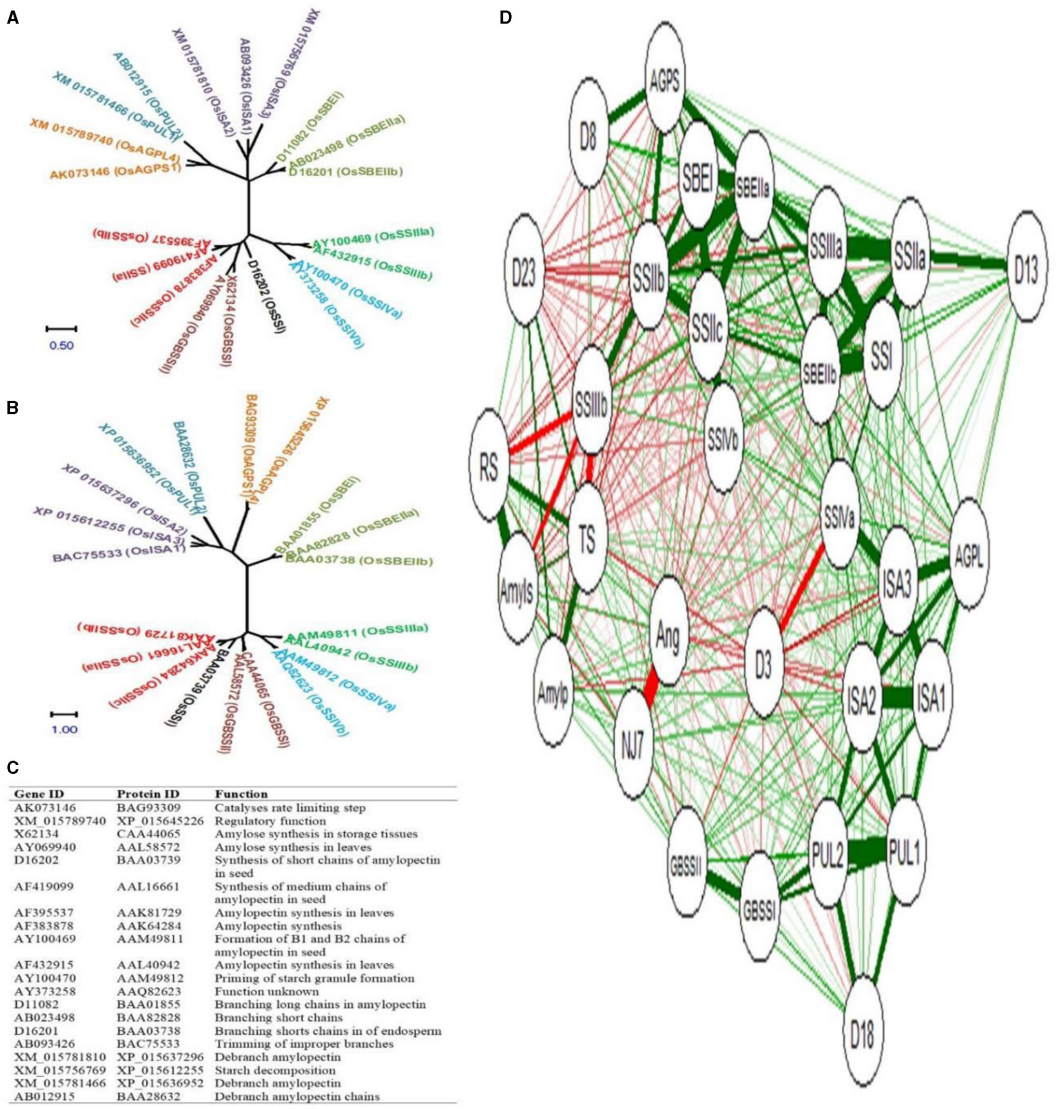
Phylogeny of starch biogenesis genes **(A)** and corresponding proteins **(B)** Gene ID, protein ID, and their catalytic functions **(C)**, and the correlation network between genes and metabolites **(D)**. Positive and negative correlations are highlighted with green and red colored edges. Color thickness is proportional to the correlation coefficient values. NJ7- NJ-72, Ang- Ananga, D3- 3DAA, D8- 8DAA, D13- 13DAA, D18- 18DAA, D23- 23DAA, RS- resistant starch, TS- total starch, Amyls- amylose, Amylp- amylopectin, AGPS- AGPS1, and AGPL- AGPL4.

The phylogenetic relationship of rice starch biogenesis genes and their corresponding protein sequences is shown in Figures 7A, B respectively. The gene ID, protein ID, and their functions are shown in Figure 7C. The rice starch biogenesis gene and protein family comprised 20 isoforms that are grouped into 9 clades: 2 AGP, 2 GBSS, 1 SSI, 3 SSII, 2 SSIII, 2 SSIV, 3 SBE, 3 ISA, and 2 PUL. Genes and proteins in the same clade conferred sequence similarities.

To simplify our understanding of the relative expression of genes and the accumulation of metabolites concerning five endosperm developing stages, the correlation network between 20 isoforms of starch biosynthesis genes and 4 metabolites (total starch, amylose, amylopectin, and RS) was developed. NJ-72 and Ananga are contrasting genotypes based on their AC and RS content, and therefore they have a strong negative correlation. The total accumulation of RS, total starch, amylose, and amylopectin was high at 23DAA and the expression level for *GBSSI, ISA1, ISA2*, and *PUL2* was high at 18DAA (Figure 7D). At 8DAA strong positive correlation was found between *AGPS1, SSIIc*, and *SBEI*. Some of the genes such as *SSIIb, SSIIIb, SSIVb, GBSSII, SBEIIa, ISA3*, and *PUL1* are not predominantly expressed in the endosperm tissue therefore their correlation was not found significant.

## 4 Discussion

### 4.1 Matrix components, microstructure, and starch quality are determinants of nutritional traits in rice

The total starch content (TSC) of 200 diverse rice genotypes in this study aligns with the findings of Omar et al. (31), who reported TSC ranging from 81.23% to 92.73% in rice. The average TSC in rice typically falls between 70% and 90%, with variations attributable to factors such as genotypic and varietal differences, and differing methods of starch estimation, including variations in the instruments used to measure absorbance. The functionality of rice starch is determined by its components, such as APC, AC, and RS, as well as the ratios between them. RS content is positively correlated with AC, with higher amylose cultivars generally exhibiting higher RS levels. Deepa et al. (32) conducted a comparative study on the RS content of pigmented (“Njavara” and “Jyothi”) and non-pigmented (“IR 64”) rice varieties, reporting RS levels between 0.6% and 1%, which is consistent with the present study's findings. Similarly, this study showed a positive correlation between RS content and AC, in agreement with the findings of Hu et al. (33), who observed that RS content increased with rising AC across three different rice cultivars. It is hypothesized that genotypes with high RS content may lower blood glucose levels by reducing the glycemic index (GI), a conclusion supported by Kumar et al. (34).

A significant positive correlation was found between the degree of crystallinity and the percentage of amylopectin. Our findings are consistent with that of Martens et al. (35) who studied starch digestion kinetics and concluded that the type of crystalline structure and amylopectin chain length distribution of starch significantly correlate with the digestion kinetics of starches across botanic sources in an *in vitro* pig model. The XRD results from this study indicate that rice genotypes with differing AC also show variations in the degree of crystallinity, which may influence starch digestibility.

Among the 10 FAs analyzed, palmitic acid, linoleic acid, and oleic acid were found in higher concentrations. Our results are consistent with Al-Bahrany (36), who examined the FAs composition in two Hassawi rice genotypes and concluded that palmitic, oleic, and linoleic acids were the most abundant. Notable, linoleic acid is the predominant polyunsaturated fatty acid (PUFA) among the studied rice genotypes. In japonica, ssIIIa mutants, linoleic acid (C18:2) was complexed with amylose to form RS5 which significantly contributes to the enhanced RS content (37). Furthermore, there is a direct correlation between the percentage of amylose and FA composition, particularly with palmitic acid, linoleic acid, and stearic acid. This amylose-lipid complex is thought to render starch more resistant to digestion. These findings are consistent with Taylor et al. (38), who reported that lipids can form complexes with amylose, subsequently reducing starch digestibility.

Protein content was highest in NJ-72 (8.98%) and lowest in RNRM-7 (7.99%). Khatun et al. (39) indicated that rice protein may affect starch digestibility by building a protective barrier around rice starch or by modifying starch characteristics, which contrast with our findings. Furthermore, Zhu et al. (40) discovered that the protein level of rice flour was inversely connected with RDS and SDS, but positively correlated with RS. Our findings matched with Li et al. (41), who studied twelve milled rice grain samples and observed that the crude protein content varies from 6.5% to 9.4%. Based on these findings, we concluded that the protein level may not significantly affect the digestibility of genotypes with varying RS levels, as there was minimal variation in protein percentage across the different genotypes.

By examining starch granule morphology, we hypothesized that high RS genotypes might be more resistant to amylase hydrolysis due to their well-packaged, complex structures and larger granule sizes. This finding agrees with Zaman and Sarbini (42), who found that smaller granules are more sensitive to enzyme digestion, attributed to their larger specific surface area, which increases enzyme binding rate. Corgneau et al. (43), reported that high RS potato starch was composed of large rounded granules having smooth surfaces. In contrast, waxy rice starch was rich in amylopectin and displayed small diameters and angular shapes. The degree of amylose, which is directly proportional to the RS, influences the size and shape of starch granules. This conclusion is supported by Krishnan et al. (44), who looked at the morphology of rice starch granules and found that genotypes with high inherent RS exhibited tight starch packing within the granules. This tight packing could contribute to the observed enzyme resistance, potentially linked to the structural complexity of amylose and amylopectin. Thus, starch granule morphology illustrates how variations in amylose percentage can influence the shape of starch granules, ultimately affecting starch digestibility and blood sugar levels.

### 4.2 Variation in metabolites at developing stages of endosperm

The maximum rate of accumulation of the carbohydrates and RS occurred between 8DAA to 23DAA. In accordance with our results Zi et al. (45) found that the rate of TSC, AC, and APC was significantly increased between 10DAA and 25DAA in developing stages of waxy and non-waxy wheat genotypes. Verma et al. (46) reported that in rice TSC was maximum at 21DAA as compared to other developing stages. Similarly, Asai et al. (47) studied the developmental changes in rice endosperm and found a rapid increase in AC up to 20 days after fertilization (DAF).

### 4.3 Alteration in activities of starch biogenesis enzymes

The activity of all the studied starch biogenesis enzymes was found higher in NJ-72 in comparison to Ananga throughout the developing stages. This difference suggests that the genes responsible for amylopectin synthesis are predominantly expressed in low amylose genotypes, such as NJ-72. At 3DAA activity of all the enzymes was very low because of the high hexose to sucrose ratio that leads to cell division rather than supplying photosynthetic carbon to endosperm development. The increased activities of SS and BE at 13 DAA in NJ-72 highlight the critical period for starch accumulation, where enzyme activity correlates with amylopectin synthesis. Furthermore, the elevated activity of pullulanase (PUL) and isoamylase (ISA) at 18 DAA suggests their roles in starch remodeling and debranching become prominent after initial primer synthesis and branching, facilitating further starch granule development. Similarly, Wang et al. (48) found maximum SS activity at 12 and SBE activity at 15 days after pollination in low and high starch-content wheat genotypes. Fujita et al. (21) studied PUL-deficient mutant rice and concluded that the function of PUL partially overlaps with that of ISA1, and the absence of PUL has a much smaller impact on the production of amylopectin than the absence of ISA1. These findings will enhance the understanding of starch biogenesis, particularly in low amylose rice genotypes, highlighting critical developmental stages where enzyme activities peak, which could inform breeding strategies for improved starch accumulation. Further, to elaborate on the differentiation in activity assays of these starch biogenesis enzymes (SS, SBE, and DBE) in developing stages of rice endosperm, genes in their respective isoforms encoding these enzymes were also evaluated.

### 4.4 Differential expression of starch biogenesis genes in the developing endosperm

Mutations in genes encoding GBSS, soluble SS, and SBE have been shown to affect RS content in rice (19, 49). The expression level of *AGPS1* in NJ-72 was slightly higher at 8DAA and 13DAA than in Ananga while in rest of the stages, *AGPS1* expression was higher in Ananga. These observations indicated that this isoform is needed for both low and high-amylose genotypes. Nagai et al. (50) studied transgenic rice expressing upregulated *cytoplasmic AGPase* in developing seeds and concluded that this gene catalyzes the rate-limiting step of the starch biosynthetic pathway. Slattery et al. (51) explored how increasing amylose levels through *AGPase* activity leads to greater RS formation and reduced starch digestibility. This suggests that manipulating *AGPS1* expression could optimize starch biosynthesis across different rice varieties and enhancing *AGPase* function could be a viable strategy for increasing RS. In contrast to our finding, Jabeen et al. (52) observed that *GBSS* protein was highly accumulated at 10DAA in contrasting GI rice lines. Downregulation of *GBSSII* at the initial stage and very low expression at later stages compared to *GBSSI* indicated that *GBSSII* may be expressed more in non-storage tissues. In addition, the expression of *GBSSI* was more at all stages compared to *GBSSII*, so it is presumed that *GBSSI* is mainly responsible for amylose synthesis and is unique to endosperm whereas *GBSSII* plays a minor role. These results are in agreement with Vrinten and Nakamura (53) who studied *GBSSI* and *GBSSII* transcripts in wheat and observed that *GBSSI* was highly expressed in the endosperm while *GBSSII* was expressed in leaf, culm, and pericarp, but not in endosperm tissue. This reinforces the notion that *GBSSII* may not be essential for starch storage in the endosperm but might play a role in other tissues, suggesting a tissue-specific regulatory mechanism. Overexpression of *GBSSI* has been also been linked to enhanced RS content in rice (54). The association of *GBSSI* overexpression with increased RS content further underscores the significance of amylose in starch digestibility and its potential benefits for developing rice varieties with favorable nutritional profiles.

Among the soluble SS, at 13DAA *SSI* showed maximum relative expression followed by *SSIIIa, SSIIa, SSIVa*, and *SSIIc*. Our findings suggest that these genes are endosperm-specific and they contribute primarily to the production of amylopectin in the NJ-72. In accordance with this, Fujita et al. (55) characterized starch biosynthesis-related enzymes in *SSI-deficient* rice mutant lines and summarized that the coordinated actions of starch synthases such as *SSI, SSIIa*, and *SSIIIa* isoforms generate amylopectin chains. Also, Hayashi et al. (56) generated the mutants of rice seeds (*ss1/ss2a/ss3a*) and concluded that mutated plants retained their capability to synthesize starch and were able to accumulate amylose but less amylopectin. Rice varieties with deleterious variants in the *SSI* gene exhibit higher RS levels, indicating the critical role of *SSI* in RS formation (57). Downregulation of *SSI* in both *japonica* and *indica* rice cultivars significantly increases AC while negatively impacting eating and cooking quality (58). In the rice cultivar Nipponbare, suppression of *SSI* alters amylopectin chain distribution, increases GBSS activity, and elevates AC (59). These findings suggest that *SSI* influences RS formation by modulating AC and amylopectin structure. Although the expression of *SSIIb, SSIIIb*, and *SSIVb* were slightly high in NJ-72 as compared to Ananga the fold variation was much less throughout the developing endosperms. In addition, their expression was minute in comparison to the above-mentioned SS genes. It can be assumed that *SSIIb, SSIIIb*, and *SSIVb* are not endosperm-specific genes (for rice) and their expression might be high in non-storage tissues.

The consistently higher expression levels of *SBE* isoforms (*SBEI, SBEIIa*, and *SBEIIb*) in NJ-72 across all developmental stages indicate a robust starch branching capability in this low amylose genotype. Wang et al. (60) used TNG82 japonica rice cultivar to mutate the *OsSBEIIb* gene through CRISPR/Cas9. The total activity of SBE at 20 days was 1.75 times higher than SBE at 25 DAF in both wild type as well as in mutant lines. The observation that *SBEIIb* had the highest expression, followed by *SBEI* and *SBEIIa*, implies a hierarchy in the functional roles of these enzymes during starch biosynthesis. *SBEIIa'*s limited role suggests that it may not be as critical for starch branching in endosperm development, which may have implications for future research targeting specific isoforms for genetic improvement. The findings are consistent with previous studies, such as that of Miura et al. (61), indicating that overexpression of *SBEI* can lead to increased RS content. This correlation suggests that manipulating the expression of *SBEI* could be a potential strategy for enhancing RS levels in rice, which is beneficial for health-related attributes such as improved glycemic response.

The overall expression of *PUL2* is higher as compared to *PUL1* in both genotypes. Therefore, it was observed that *PUL2* is more important than *PUL1* as a DBE. Among the 5 DBEs at 18DAA, *PUL2* has the highest expression followed by *ISA1* and *ISA2*. Similarly, Yamakawa et al. (62) studied the expression of grain-filling-related genes in rice at 10DAF under high temperatures and based on microarray and RT-PCR results reported that *ISA1, ISA3*, and *PUL* showed 0.94, 0.93, and 0.83-fold change respectively. In contrast to their findings, our results indicated that *PUL2* was predominantly expressed followed by *ISA1*, while *ISA3* having minor role as a DBE. Ohdan et al. (10) reported that all the DBEs had low levels of transcripts at the early stages of seed development (1-3 DAF), which was maintained by both *ISA2* and *ISA3* up to the later stages. But *PUL* and *ISA1* showed 12 and 56-fold increases in transcript level from 3 to 7 DAF respectively advising that *ISA1* and *PUL* play crucial roles in the starch accumulation process throughout the endosperm. In addition to their findings, the current results also indicated that the 18DAA stage is the most favorable stage for studying the expression of DBEs and at this stage, structural winding and unwinding of starch molecules occurs the most. This knowledge can guide future research to optimize the timing of interventions aimed at improving starch characteristics. *PUL* plays a role in RS formation by releasing linear amylose-like chains that promote starch retrogradation, resulting in the synthesis of type 3 RS and reduced starch digestibility (63). This aspect is crucial for developing rice varieties with enhanced nutritional benefits, especially for consumers seeking low GI foods.

### 4.5 Gene to metabolite correlation and phylogeny

Among the metabolites, RS and AC have a strong positive correlation (r = 0.96). Krishnan et al. (44) in rice varieties showed that RS is moderately dependent on the percentage of amylose while weakly dependent on the percentage of amylopectin. This reinforces the importance of focusing on amylose synthesis and regulation to influence RS content in rice varieties. In comparison to other starch biogenesis genes, GBSS has a slightly positive correlation with AC (r = 0.52) and RS (r = 0.40), while with amylopectin it has a weak positive correlation (r = 0.28). Similarly, Zi et al. (45) reported that AC was found to be significantly and positively correlated with GBSS activity (r = 0.80) in waxy and non-waxy wheat cultivars. SS, DBE, AGP, and BE were found to be correlated with the RS content to some extent in the rice-developing grains and their weaker relationships suggest that *GBSS* may be more critical for enhancing RS. This could mean that manipulating GBSS expression may yield more significant improvements in RS content compared to other genes. At 13DAA there is a strong positive correlation between *AGPL4, SSI, SSIIa, SSIIIa, SSIVa*, and *SBEIIb*. Overall results showed strong networking of starch biogenesis genes with metabolites in starch accumulation and structural partitioning in the form of AC, APC, and RS at differential levels during the developmental stages of rice endosperm.

## 5 Conclusion

Rice starch digestibility depends on several factors, including the amount of amylose, amylopectin, RS, compositions of different FAs, starch granule morphology, crystallinity degree percentage, protein percentage, the enzymatic activity of starch biogenesis, etc. Amylose has a significant positive correlation with that of RS and compactness of starch, but it has a negative correlation found with the percentage of amylopectin, crystallinity degree percentage, activity of SS, SBE, and ISA, etc. As the amount of amylose increases, it causes tight packaging of starch granules and a low crystallinity degree percentage that might limit the availability of starch to the digestive enzymes and hence lowers the energy density. Among the metabolites, RS and AC have a strong positive correlation with their corresponding encoding genes. The present study also revealed that a positive correlation exists between starch biosynthetic enzyme activity and AC and TSC accumulation in two contrasting rice genotypes. The genes exhibiting high expression either at early or late or across all the endosperm developmental stages can be used as a candidate gene for developing molecular markers through association or linkage analysis, which further can be used for starch quality improvement in rice.

Therefore, these findings suggest that eating rice that has high amylose and/or RS may lower blood glucose levels and hence reduce the risk of diseases resulting from high blood sugar. It can be utilized to modify the starch biogenesis enzymes and genes to meet the requirement of quality starch for increasing population, and expanding food industries while keeping in mind to protect the environment from non-degradable biopolymers. Further investigations are required to reveal how the alterations in the enzymatic activities related to starch biosynthesis govern the partitioning of AC, APC, and RS affecting structural modulations toward the starch granule formation and accumulation of quality starch contents (RS).

## Data Availability

The original contributions presented in the study are included in the article/Supplementary material; further inquiries can be directed to the corresponding author.
