# Supplementary figures and images for: Exploring the synergy of enzymes, nutrients, and gene networks in rice starch granule biogenesis
Source: Front Nutr. 2024 Oct 23;11:1448450. doi: 10.3389/fnut.2024.1448450 (PMC11538003; doi:10.3389/fnut.2024.1448450)

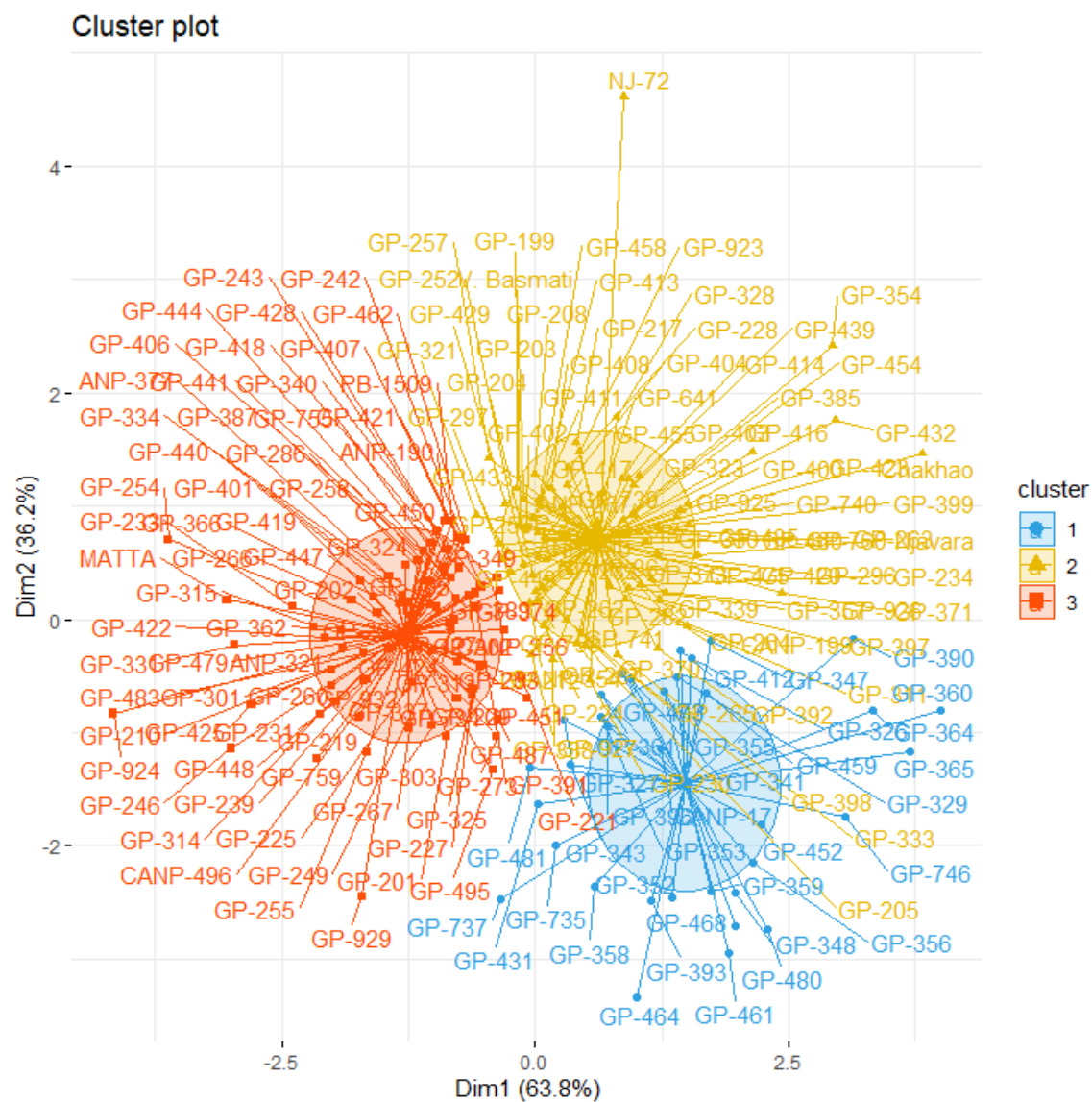

FIGURE S1. Clustering of 200 rice genotypes based on percentage of amylose content

Supplement: Supplementary file 1 [file Image_1.pdf]
